# The West Africa Lassa fever Consortium pre-positioned protocol for a Phase II/III adaptive, randomised, controlled, platform trial to evaluate multiple Lassa fever therapeutics

**DOI:** 10.12688/wellcomeopenres.19041.2

**Published:** 2023-06-02

**Authors:** Josephine Bourner, Alex Paddy Salam, Marie Jaspard, Adebola Olayinka, Camille Fritzell, Bronner Goncalves, Michel Vaillant, Tansy Edwards, Cyril Erameh, Nnennaya Ajayi, Michael Ramharter, Piero Olliaro, The WALC Work Package 2 Working Group

**Affiliations:** 1 Pandemic Sciences Institute, University of Oxford, Oxford, UK; 2 University of Bordeaux, Bordeaux, France; 3 The Alliance for International Medical Action, Dakar, Senegal; 4 Nigeria Centre for Disease Control, Abuja, Nigeria; 5 Competence Center for Methodology and Statistics, Luxembourg Institute of Health, Luxembourg, Luxembourg; 6 The London School of Hygiene and Tropical Medicine, London, UK; 7 Irrua Specialist Teaching Hospital, Irrua, Nigeria; 8 Alex Ekwueme Federal University Teaching Hospital, Abakaliki, Nigeria; 9 Bernhard Nocht Institute for Tropical Medicine, Hamburg, Germany; 10 Dept of Medicine, University Medical Center Hamburg-Eppendorf, Hamburg, Germany

**Keywords:** Lassa fever; pre-positioned protocol; Phase II/III; clinical trial

## Abstract

**Background**: This is a standardized, pre-positioned protocol for the coordinated evaluation of Lassa fever therapeutics. The protocol is the product of discussions that took place in 2021 and 2022 among international investigators from a wide range of scientific and medical disciplines working together within the West Africa Lassa fever Consortium (WALC).

**Methods**: This is a clinical Phase II/III multicentre randomised controlled platform trial using a superiority framework with an equal allocation ratio and a composite primary endpoint of all-cause mortality OR new onset of i) acute kidney failure (AKF), OR ii) acute respiratory failure (ARF), OR iii) shock assessed from enrolment (D0) to D28.

**Discussion**: This pre-positioned protocol was developed by the WALC and made available for adaptation and implementation by the wider Lassa fever research community in order to generate efficient, reliable, and comparable evidence for Lassa fever therapeutics.

## Administrative information

### Guidance for researchers using this pre-positioned protocol

A pre-positioned protocol is a proposed protocol for a clinical trial. Its authors have no present intention to implement the trial at the time of publication of this article. The intention of this pre-positioned protocol is to share a potential trial design that has been developed among the Lassa fever research community in order to harmonise clinical trial activities. Research groups who wish to implement this protocol may use it exactly as described herein or make adaptations to the proposed design. 

This pre-positioned protocol has been structured according to the SPIRIT template ^1, 2^. The brackets {} included in the headings indicate the corresponding SPIRIT checklist item. The text included in this pre-positioned protocol contains the minimum information set for the study described. Modifications may be required based on: 1) the setting in which the protocol is implemented to comply with local regulations around the conduct of clinical trials; 2) the operational requirements of the study.

Boxes include guidance on the additional information that may be required ahead of a protocol being submitted to regulatory authorities or ethics committees for review and some recommendations for additions or adaptations to the eventual design. These boxes help identify context-specific adaptations and provide information to help investigators decide on study design options.

If you make modifications/translations/improvements we would be grateful if you would consider sharing these - however minor they are - with the international community through WALC ( research@isaric.org).

Note that the wording in the sections titled ‘Consent and assent’, ‘Adults who lack the capacity to provide informed consent’, ‘Adults who are unable to read’, ‘Participant withdrawal’, ‘Data management’ and ‘Definition of adverse events’ is based on the wording provided in the clinical trial protocol templates developed by the University of Oxford.

### Open-source license

This pre-positioned protocol was created by members of WALC (West Africa Lassa fever Consortium) and is distributed under the Creative Commons Attribution Non-commercial ShareAlike Licence version 4.0 ( http://creativecommons.org/licenses/by-nc-sa/4.0). It is freely available for you to copy, adapt, distribute and transmit under the conditions that: a) the original source is attributed; b) the work is not used for commercial purposes; c) any altered forms of this document are distributed freely under the same conditions.

### Pre-positioned protocol version {3}

Version 1.0 date 04 Aug 2022

## Introduction {6a}

This is a standardized protocol for the coordinated evaluation of Lassa fever therapeutics. The protocol is the product of discussions that took place in 2021 and 2022 among an international group of investigators from a wide range of scientific and medical disciplines working together within the West Africa Lassa fever Consortium (WALC). The WALC is a collaboration, rooted in West Africa, between a broad range of stakeholders with the objective of outlining clinical development pathways for the successful development of Lassa fever therapeutics. One objective of the consortium was to develop and agree upon a methodology for the evaluation of Lassa fever therapeutics in Phase II and III clinical trials – the output of which is described here in this protocol. Surrounding this objective were other complementary objectives addressed by the WALC that aim to address capacity strengthening for clinical trials in West Africa, develop a Target Product Profile for novel therapeutics and consider an end-to-end framework for the development, manufacturing and availability of effective drugs in Lassa-endemic countries. The outputs of these objectives are described elsewhere ^3^.

### Background and rationale

Lassa fever is an acute haemorrhagic disease caused by the Lassa virus. Although the virus has been found in a number of rodent species, *Mastomys natalensis* is the primary reservoir and, once infected, is able to shed the virus through urine and droppings ^4, 5^. While *M. natalensis* can be found throughout Sub-Saharan Africa, the virus is endemic to West Africa, where it is estimated to cause up to 300,000 new clinical cases per year ^4, 6, 7^.

The highest regional burden of Lassa fever is found in Nigeria where, in 2020 (the last year for which data was available on the Nigeria Centre for Disease Control website), a total of 1181 confirmed cases were detected which resulted in 242 (20%) deaths ^8^. Cases are also reported annually in Sierra Leone ^9^ and Liberia ^10^, with more sporadic events reported in Guinea ^11^, Benin ^12^ and Togo ^13^. There are several specialist Lassa fever treatment centres in Nigeria ^14^, of which those in Edo, Ondo, Ebonyi and Bauchi States see ~75% of all cases ^15^, and one in Sierra Leone ^9^; health facilities of varying sizes and capacities in other countries occasionally receive cases of Lassa fever as they arise ^3^.

Transmission to humans occurs primarily through contact with contaminated surfaces, but human-to-human and laboratory transmission can also occur ^16^, particularly in low-resource settings with sub-optimal infection prevention and control practices ^17^. Healthcare workers, pregnant women and children are at considerable increased risk of both infection and poor outcomes ^18, 19^.

Onset of Lassa fever symptoms usually occur within 6 – 21 days of infection and are typically characterised by non-specific symptoms, such as fever, headache, vomiting, diarrhoea and muscle pain ^16^. In severe cases, symptoms can include acute kidney injury, shock, haemorrhage and encephalopathy ^16^. Lassa fever is estimated to cause approximately 5,000 deaths per year with a case fatality rate (CFR) between 12% – 30% for hospitalised cases and approximately 1–2% overall ^6, 20, 21^. 

No drug has yet received regulatory approval for the treatment of Lassa fever. Ribavirin is currently used off-licence in conjunction with supportive care ^22^. Evidence for this treatment recommendation derives from results of a study conducted in the 1980s ^23^; no further clinical trials to assess Lassa fever therapeutics, including ribavirin, have been conducted. Concerns however have been raised about lack of rigorous randomised trial data to inform treatment guidelines and reliability of the trial performed in 1980s due to the limitations of the study design ^24^. A recent reanalysis of the data suggests increased mortality in patients who received ribavirin with serum AST <150 IU/L ^25^. There is therefore an urgent need to evaluate other therapeutic options.

Here we present a pre-positioned clinical trial protocol for a Phase II/III multi-centre randomised controlled platform trial.

Given the small number of potential trial sites – in the interest of conducting a cost-effective and efficient trial, the five sites in the areas of Nigeria and Sierra Leone that see the highest incidence of Lassa fever would likely be prioritised – and patients who can be enrolled throughout the Lassa-endemic areas, combined with the prospect of having a number of potential treatment candidates to test, the customary approach of individual separate trials will be inadequate and ineffective, thus requiring a pre-positioned platform trial design.

We selected a platform trial design approach as there are a number of drug candidates that are ready, or will shortly be ready, for evaluation: one existing antiviral drug (favipiravir) that could potentially be repurposed for Lassa fever, and three experimental drug candidates. Of the three, one is in phase I clinical development (LHF-535, small chemical, under US Investigational New Drug (IND) application), and two are in advanced pre-clinical development nearing US IND application (one small chemical, ARN-75039, and one monoclonal antibody, Arevirumab-3) ^26^. Evaluating these drugs under a single protocol has several advantages, including: ensuring the harmonisation and comparability of results and conditions of testing; efficiency in using a limited Lassa fever patient population; accelerating the generation of results; and cost-effectiveness from using a shared clinical trial infrastructure. Platform trials also have the advantage of providing a framework in which new treatment arms can be added as new drugs become available, or treatment arms of combination regimens.

A combined Phase II/III approach will also allow the gathering of consistent coherent information on the disease and to test drugs using the same parameters across phases.

## Methods

### Overview

**Figure 1.**
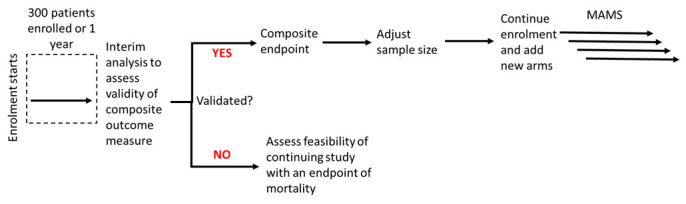
Trial design. MAMS: Multi-Arm Multi-Stage Trial design.

This is a Phase II/III multicentre individually randomised controlled platform trial using a superiority framework with an equal allocation ratio and a composite primary endpoint of all-cause mortality OR new onset of i) acute kidney failure (AKF), OR ii) acute respiratory failure (ARF), OR iii) shock assessed from enrolment (D0) to D28 (see Figure 1 for an illustration of the trial design and Table 3 for the definition of the components of the clinical outcome).

### Objectives {7}

***Primary objective***

The primary objective of this protocol is to evaluate the safety, tolerability and efficacy of new Lassa fever therapeutics.

***Secondary objectives***

The secondary objective of this protocol is to evaluate the frequency of complications associated with Lassa fever.

***Other study objectives***

**Additional information required:**

(i) Add information on whether PK/PD studies are going to be performed in parallel;

(ii) Add information on whether additional characterisation of the infection, either on the host or virus, will be performed.

## Trial design {8}

### Choice of endpoints

As is the case for many emerging infectious diseases, clinical research on Lassa fever faces several challenges, including fluctuating annual case numbers which are heavily influenced by climate, and difficult field conditions. These different factors, that limit the capacity to recruit study participants, together with the number of mortality events that would be too small to detect significant differences between treatment arms, which has implications to the study sample size, impact the feasibility of clinical trials. Where a clinically relevant composite endpoint can be agreed that reflects unfavourable outcome, the risk of this outcome will be greater than the risk of mortality alone. A higher risk of unfavourable outcome will correspond to a smaller sample size requirement to detect differences between arms, compared to the sample size requirement when using mortality as endpoint. Another advantage of using a clinically relevant composite outcome relates to improved external validity of the trial results. Availability of medical resources for supportive care, which likely influences risk of mortality, varies considerably in areas where Lassa fever is endemic; by including as components of the composite outcome clinical conditions such as acute kidney failure and shock, treatment effects observed in the trial would capture improved survival in settings with limited resources (e.g. where dialysis is not available). 

Since only very limited data are available to inform the frequencies of components of a composite outcome both at admission and after hospital admission, this protocol uses an innovative design approach that first assesses the validity and frequency of a composite outcome to allow the use of a more frequent endpoint, compared to the death outcome. The protocol will also allow the concurrent assessment of multiple therapeutic options, after the initial validation of the composite endpoint, to effectively use the limited case numbers. This approach will provide the most efficient means of generating the evidence required to find an optimal treatment regimen for Lassa fever.

### Choice of comparators {6b}

Although unlicensed, ribavirin, used off-label, in addition to supportive care is the only recommended treatment for Lassa fever and will therefore be used as the control intervention in this study. In particular, we investigated the acceptability of use of placebo in the comparator group in the West Africa Lassa fever Consortium (WALC) and, following consultation with local clinicians (through surveys and discussions that took place within the working group), it was determined that the use of placebo would not be well-supported at participating sites and therefore would not be feasibly incorporated under a trial protocol. The WALC found that 60% of the doctors involved in the consultation stated they would not be willing to enrol patients in a study in which some patients would receive supportive care alone.

For the treatment interventions, in addition to detailed information on the drugs that will be included in the initial comparison of the trial, information on future treatment arms will be presented in amendments of this protocol, as annexes. Candidate therapeutics that are currently progressing through the R&D pipeline for Lassa fever and could be included in the interventional arm include favipiravir ^27^, LHF-535 ^28^, ARN-75039 ^29^, and Arevirumab ^30^.

**Additional information required:**

(i) Add information of chosen ribavirin regimen and references to the evidence supporting the choice of regimen;

(ii) Add information on the drugs that will be included in the first comparison;

(iii) Information on the therapies that will be added to the trial after recruitment starts should be included as annexes to the protocol.

### Initial interim analysis {21b}

The trial will involve at least one interim analysis to assess the validity and frequency of the composite outcome and allow for sample size re-estimation. Due to the uncertainty around the frequencies of the events included in the composite endpoint – due to the highly variable prevalence of the events reported in the existing academic literature – the trial will initially be powered for an all-cause mortality endpoint, which may require a large sample size. After 300 participants are recruited or after one year of recruitment, whichever comes first, an analysis of the validity of the composite outcome will be performed. The focus of this analysis will be to describe the prevalence of the component events of the composite endpoint at the time of randomisation. If the composite outcome is deemed valid, the frequency of the composite endpoint will be used to re-estimate the required sample size. If, in this initial interim analysis, it is shown that the sample size can be feasibly achieved using the composite endpoint, the sample size will be adjusted via an amendment and the trial will continue with the composite endpoint as the primary outcome measure. If it is shown that composite outcome is not a valid endpoint (e.g. most study participants are diagnosed with multiple components of the composite outcome on admission) or that its frequency is not considerably higher than the frequency of death outcome in the study population, the trial team will consider the feasibility of continuing the study with a mortality endpoint and make the relevant amendments to the protocol (see additional information on the sections on sample size and interim analyses).

The assessment of the validity of the composite outcome measure, to be conducted by an independent panel, will be based on:

- The proportion of patients enrolled in the trial who present with one or more of the components of the composite endpoint on admission
- The proportion of patients experiencing a new event, defined as one of the components of the composite outcome, following admission
- The variation of the frequency of the events by study site and over time, as the profile of patients recruited to the trial and the time from infection to hospital admission might change.

**Suggestions:**

(i) Note that whilst we suggested that the analysis to assess the validity of the composite outcome is performed after 300 patients are recruited, which corresponds approximately to the expected number of patients recruited in one year, the trial team might choose a different approach, for example related to desired level of precision for the frequency of outcome. The rationale for this relatively high number of patients recruited before the initial interim analysis relates to the seasonality of Lassa fever incidence (if interim analysis can be performed just after the first transmission season) and to having sufficient data before initial analysis to reduce the impact of initial temporal changes that might happen in the recruitment of patients (e.g. patients might present earlier in the course of their disease as recruitment progresses, due to increased awareness).

(ii) In addition to the sample size re-estimation, the trial team will have three options to consider:

(a) no interim analyses using efficacy or futility stopping rules are performed;
(b) efficacy stopping rule is used with the primary endpoint after validity of composite outcome is assessed;
(c) futility stopping rule is used with the primary endpoint after validity of composite outcome is assessed. Considerations on the use of stopping rules are presented in the Sample Size section.

Figure 1 below illustrates the design, where an initial group of patients will be recruited (for example, here we considered an initial recruitment of 300 patients or one year, whichever comes first). The validity of the composite outcome will be assessed during this initial analysis and sample size might be re-estimated. After establishing that the composite outcome can be used as the primary outcome, additional treatment arms can be added to the study (see below). 

### Inclusion of new study arms

The initial analysis to establish the validity of the composite outcome is essential for the conduct of the trial. After establishing the composite outcome can be the primary endpoint of the trial, the inclusion of new arms to be compared to the control arm will be possible. The protocol will follow the approach used in the Pamoja Tulinde Maisha (PALM [“Together Save Lives” in the Kiswahili language]) trial, for Ebola virus disease treatment ^31^. In that trial, control of the type I error in the analysis did not account for multiple pairwise comparisons (each treatment arm versus control arm). Many of the arguments used to justify that decision are valid for Lassa fever trials, in particular: high mortality in hospitalised patients, including outside study settings; intermittent epidemics, which implies uncertainties in patient recruitment; and urgency to identify effective therapies. Furthermore, methodological work suggests control of family-wise error rate in trials involving multiple pairwise comparisons to a single control arm is not essential if the different treatment arms being compared do not generate evidence that will support the approval of a single class of drugs ^32, 33^. The timing of the inclusion of new arms will depend on considerations on its impact on the recruitment to the existing arms and on the management of the trial and its logistics.

## Study setting {9}

This is a multicentre trial taking place in tertiary-level health centres in West Africa.

**Additional information required:**

Add information on the study sites, including if available information on expected number of patients with Lassa fever admitted per year.

## Study population and eligibility criteria {10}

This trial will enrol adult, non-pregnant hospitalised patients with Lassa fever at participating health centres in West Africa. 

A blood sample will be taken from potential participants for laboratory confirmation of Lassa fever by reverse transcriptase polymerase chain reaction (RT-PCR) as part of routine care according to local guidelines. The sample used for confirmation of Lassa fever (LF) will be taken before enrolment in the study.

### Inclusion criteria

All patients included in the trial must meet the following criteria:

- RT-PCR confirmation of LF
- Adult participants (defined as a person who has attained the age of majority according to national regulations in their country of enrolment)

Note: Depending on the drugs being tested, women of childbearing potential (WOCBP) must have a negative pregnancy test in order to participate in the study (see section on inclusion of women of childbearing potential). 

### Exclusion criteria

Patients will be excluded from the trial if they meet any of the following criteria:

- Patients receiving end-of-life care for another illness
- Involvement in another clinical trial
- Unwilling to provide informed consent
- History of allergic reaction or other contra-indication to trial drugs
- Received drug therapy for Lassa fever (excluding supportive care) prior to inclusion

**Additional information required:**

Add other exclusion criteria based on drug profile and trial design.

### Inclusion of women of childbearing potential

WOCBP are defined as fertile, following menarche and until becoming post-menopausal unless permanently sterile. Permanent sterilisation methods include hysterectomy, bilateral salpingectomy and bilateral oophorectomy. A postmenopausal state is defined as no menses for 12 months without an alternative medical cause. The inclusion of WOCBP requires use of a highly effective contraceptive measure following a negative pregnancy test.

Acceptable contraceptive measures include:

•   Combined (estrogen and progestogen containing) hormonal contraception associated with inhibition of ovulation:

○   Oral

○   Intravaginal

○   Transdermal

•   Progestogen-only hormonal contraception associated with inhibition of ovulation:

○   Oral

○   Injectable

○   Implantable

•   Intrauterine device (IUD)

•   Intrauterine hormone-releasing system (IUS)

•   Bilateral tubal occlusion

•   Vasectomised partner

•   Sexual abstinence

Contraception should be maintained during treatment and until the end of relevant systemic exposure period.

At the end of the systemic exposure period, a final urine/serum pregnancy test must be taken by all enrolled WOCBP.

### Inclusion of male partners of women of childbearing potential

Male participants are considered fertile after puberty unless permanently sterile by bilateral orchiectomy.

All fertile male participants meeting the above definition should use condoms during treatment and until 90 days after the end of relevant systemic exposure.

## Interventions {11a}

This is a multi-arm platform trial and will allow the comparison of multiple investigational treatment regimens. Participants randomised to the control arm will receive ribavirin. Information on the drugs tested in the initial comparison is presented below; information on therapies that will be included later in the trial will be included as annexes of the protocol.

**Additional information required:**

(i) Add details of all regimens (including regimens to be used in sub-populations e.g. pregnant women, where applicable) for all drugs included in the protocol that will be used in the initial comparison.

(ii) For any drugs added to the study after study initiation, include this information in annexes of the protocol, as amendments.

### Modifications {11b}

For a given trial participant, the assigned study intervention may need to be modified or discontinued by trial investigators for various reasons, including as a result of adverse events or withdrawal of consent.

**Additional information required:**

Describe criteria for modifications and discontinuations

Regardless of any decision to modify or discontinue their assigned intervention, study participants should be retained in the trial whenever possible to enable follow-up data collection and prevent missing data.

### Adherence {11c}

Patients enrolled in this study will be inpatients and their treatment will be administered directly by the research team. Information on drug, dose and timing of dosing will be recorded on the eCRF and monitored throughout the study.

### Supportive care

All patients enrolled in the trial will receive supportive care. However, it may not be feasible to standardise the supportive care available to all patients enrolled across participating sites, as the availability of treatment and equipment is highly variable and requires significant investment and capacity strengthening that extends beyond the scope of this trial.

A minimum standard of supportive care will therefore be defined in this protocol up until the point that an intervention is required or the trial endpoint is met. At this point, the treating clinician will be responsible for deciding the onward management of the patient.

All other supportive care will be at the discretion of the treating clinician.

### Acute Kidney Injury

The supportive care defined below follows the KDIGO Clinical Practice Guideline for Acute Kidney Injury (AKI) ^34^.

AKI is defined as:

- Increase in Serum Creatinine (SCr) by ≥0.3 mg/dl (≥26.5 lmol/l) within 48 hours; or
- Increase in SCr to ≥1.5 times baseline, which is known or presumed to have occurred within the prior 7 days; or
- Urine volume <0.5 ml/kg/h for 6 hours

SCr should be measured at least every 72 hours for the first two weeks of hospitalisation. Sites should have the capacity to monitor urine output via urinary catherization as needed.

AKI should be staged according to the KDIGO criteria ( Table 1) ^34^

**Table 1.** KDIGO AKI staging criteria.

Stage	Serum Creatinine	Urine output
1	1.5–1.9 times baseline OR ≥0.3 mg/dl (≥26.5 mmol/l) increase	<0.5 ml/kg/h for 6–12 hours
2	2.0–2.9 times baseline	<0.5 ml/kg/h for ≥12 hours
3	3.0 times baseline OR Increase in serum creatinine to ≥4.0 mg/dl (≥353.6 mmol/l) OR Initiation of renal replacement therapy OR, In patients <18 years, decrease in eGFR to <35 ml/min per 1.73 m2	<0.3 ml/kg/h for ≥24 hours OR Anuria for ≥12 hours

This protocol recommends the following treatment for AKI:

- Isotonic crystalloids should be used in the initial management of AKI
- Diuretics should only be used in the management of volume overload
- Shock should be managed according to the section on Shock below

**The decision to start dialysis will be at the discretion of the treating clinician.**

Clinicians should aim to achieve a total energy intake of 20–30 kcal/kg/d in patients with any stage of AKI, with nutritional supplements or nasogastric feeding as appropriate

### Respiratory Failure

The supportive care described below has been informed by the Scandinavian clinical practice guideline on fluid and drug therapy in adults with acute respiratory distress syndrome ^35^.

Respiratory failure is defined as oxygen saturation <92%.

Oxygen saturation should be monitored at least 3 times per day using a pulse oximeter. Target oxygen saturation should be 92%.

This protocol recommends the treatment of respiratory failure via:

- Oxygen support (through any available method) should be given to patients when oxygen saturation reaches ≤92%
- A restrictive fluid therapy should be considered in patients with respiratory failure secondary to pulmonary oedema or volume overload

**Once the patient has SpO2/FiO2 ≤ 315, all onward care is at the discretion of the treating clinician, including the decision to initiate non-invasive or mechanical ventilation.**

### Shock

The supportive care described below has been informed by the Recommendations for sepsis management in resource-limited settings ^36^.

Shock is defined as Mean Arterial Pressure <65 mm Hg.

Temperature, heart rate, oxygen saturation and blood pressure should be monitored at least 3 times daily. Target MAP should be ≥65 mm Hg. Urine output should be monitored ideally with a urinary catheter.

This protocol recommends the following treatment for shock:

- Patients in shock should receive broad-spectrum antibiotics
- Use crystalloids for fluid resuscitation
- Noradrenaline is the vasopressor of choice but adrenaline or dopamine can be used if noradrenaline is not available
- Steroids are not routinely recommended in patients with Lassa fever who are hypotensive

### Encephalopathy

Encephalopathy is defined as an altered level of consciousness with or without the presence of seizures ^37^.

The patient’s consciousness should be assessed at least three times per day using the ACVPU scale.

This protocol recommends the following treatment for encephalopathy:

- Diazepam should be given as the first-line treatment for seizures
- Broad-spectrum antibiotics (ceftriaxone) should be given to all patients with signs of meningism, focal neurology or seizures

### Bleeding

All patients should be monitored for signs of bleeding. Hb should be measured at least every 72 hours during the first 14 days of hospitalisation.

All sites should have the ability to monitor clotting parameters.

All patients should be graded according to the WHO Bleeding Scale ( Table 2).

**Table 2.** WHO bleeding scale.

Grade	Examples
2	• Epistaxis, with the total duration of all episodes over 30 minutes in 24 hours. • Purpura over 2.5 cm (1 inch) in diameter. • Joint bleeding. • Melanotic stool. • Haematemesis. • Gross/visible haematuria. • Abnormal vaginal bleeding (more than spotting). • Haemoptysis. • Visible blood in body cavity fluid. • Retinal bleeding without visual impairment. • Bleeding at invasive sites.
3	• Bleeding needing red blood cell transfusion over routine transfusion needs. • Bleeding associated with moderate haemodynamic instability.
4	• Bleeding associated with severe haemodynamic instability. • Fatal bleeding. • Central nervous system bleeding on imaging study with or without dysfunction

**Table 3.** Composite primary endpoint.

Parameter (any of the following):	Measurement definition	Assessment timepoint
Death	Y/N	Day 0 - 28
New onset of acute kidney failure ^a^	KDIGO 3 ^b^	D0 – discharge from hospital
New onset of acute respiratory failure ^a^	SpO _2_/FiO _2_ ≤ 315 Based on 2 consecutive measurements taken at least 4 hours apart meeting the above criteria	
New onset of shock ^a^	MAP < 65 mmHg Based on 2 consecutive measurements taken at least 4 hours apart meeting the above criteria	

^a^ The composite endpoint assesses the new onset of an event from the point of inclusion. ^b^ 3.0 times baseline, OR increase in serum creatinine to ≥4.0 mg/dl (≥353.6 mmol/l), OR initiation of renal replacement therapy ^21^.

This protocol recommends that a blood transfusion should be provided to patients with Hb <8 g/dL.

## Outcomes {12}

### Primary outcome measure

The trial will evaluate the proportion of patients meeting any one of the components of the below composite endpoint ( Table 3):

### Secondary outcome measures

The trial will evaluate the secondary outcome measures described in Table 4.

**Table 4.** Secondary outcome measures.

Parameter	Definition	Measurement	Timepoint(s)
All-cause mortality	Y/N	Number of participants with mortality by D14	D0 - D14
Acute kidney failure	KDIGO 3	Number of participants meeting KDIGO 3 by D14	D0 - D14
Acute respiratory failure	SpO _2_/FiO _2_ ≤ 315 Based on 2 consecutive measurements taken 4 hours apart meeting the above criteria	Number of with SpO _2_/FiO _2_ ≤ 315 by D14	D0 - D14
Shock	MAP < 65 mmHg Based on 2 consecutive measurements taken 4 hours apart meeting the above criteria	Number of participants with MAP <65 mmHg by D14	D0 - D14
New onset of severe anaemia	Hb level <80 g/L ^40^	Number of participants meeting the definition of ‘severe anaemia’ by D14 and by D28	D0 - D14 D0 - D28
Viral clearance	Time in Days to first negative Lassa Virus RT-PCR in blood	Median number of days to RT-PCR negativity	D0 - D28
Hearing loss	CTCAE Grade 3 or above “hearing impaired” ^41^	Number of participants meeting the criteria for CTCAE Grade 3 or Grade 4 hearing impaired at D14 and D28	D0 – discharge from hospital
Bleeding	WHO bleeding scale Grade 2 or above	Number of participants who have had a bleeding event meeting WHO bleeding scale Grade 2 or above	D0 - D14
Encephalopathy	Altered level of consciousness with or without the presence of seizures	C or below on ACVPU	D0 - D14 D0 - D28
Number of days of hospitalisation beyond the end of treatment	Time in days from end of treatment to hospital discharge	Median number of days from end of treatment to hospital discharge	D0 - D28
Duration of Renal Replacement Therapy (RRT)	Time in days from initiation of RRT to end of RRT	Median number of days from initiation of RRT to end of RRT	D0 - D28
Duration of oxygen therapy	Time in days from initiation of oxygen therapy to end of oxygen therapy	Median number of days from initiation of oxygen therapy to end of oxygen therapy	D0 - D28
Pregnancy outcome	i) Ectopic pregnancy, ii) Spontaneous abortion, iii) Elective termination (foetal defects), iv) Elective termination (foetal defects or unknown), v) Stillbirth with foetal defects, vi) Stillbirth without foetal defects, vii) Live birth with congenital anomaly, viii) Live birth without congenital anomaly	Number of patients with i) Ectopic pregnancy, ii) Spontaneous abortion, iii) Elective termination (foetal defects), iv) Elective termination (foetal defects or unknown), v) Stillbirth with foetal defects, vi) Stillbirth without foetal defects, vii) Live birth with congenital anomaly, viii) Live birth without congenital anomaly	At pregnancy outcome
Preterm births	Y/N	Number of live births born preterm	At birth
Neonatal size	Live births born small-for-gestational age (SGA)	Number of live births born small-for-gestational age (SGA)	At birth
Birthweight	Live births with low birthweight (LBW)	Number of live births with low birthweight (LBW)	At birth
Neonatal death	Y/N	Number of neonatal deaths from birth (D0) to D28	Up to 28 days after birth
Adverse events	Number of adverse events	Number of participants experiencing an adverse event on or before D14 and D28	D0 - D14 D0 - D28
Serious adverse events	Number of serious adverse events	Number of participants experiencing a serious adverse event on or before D14 and D28	

## Participant timeline {13} 

The schedule of events is described in Table 5.

**Table 5.** Schedule of events.

	STUDY PERIOD														
	Enrolment	Allocation	Treatment											Follow-up	
TIMEPOINT**	*-t _1_^f^*	*0 ^f^*	*t _1_*	*t _2_*	*t _3_*	*t _4_*	*t _5_*	*t _6_*	*t _7_*	*t _8_*	*t _9_*	*t _10_*	*t _x_*	*D14*	*D28*
**ENROLMENT**															
**Screening**	X														
**Informed consent**	X														
**Randomisation**		X													
**Demographics**		X													
**Comorbidities**		X													
**Signs and symptoms**		X	X	X	X	X	X	X	X	X	X	X	X	X	X
**HIV RDT**		X													
**Malaria RDT**		X													
**Pregnancy test**		X											X		
**Concomitant medication**		X	X	X	X	X	X	X	X	X	X	X	X	X	X
**INTERVENTIONS:**															
**Ribavirin**			X	X	X	X	X	X	X	X	X	X	X		
***Intervention B ^a^***															
***Intervention C ^a^***															
**ASSESSMENTS:**															
**Vital signs ^b^**		X	X	X	X	X	X	X	X	X	X	X	X	X	X
**Audiometry**		X												X ^g^	X
**Blood sample for hematology ^c^**		X			X		X			X			X	X	
**Blood sample for biochemistry ^d^**		X		X		X		X		X		X			
**Urinalysis ^e^**		X													
**Blood sample for RT-PCR**		X				X			X			X		X	X
**Adverse events**		X	X	X	X	X	X	X	X	X	X	X	X	X	X

^a^ *Interventions and timepoints at which they are administered to be added as they become available* ^b^ Performed 3 times daily (see Section 9.1.4) ^c^ Including haemoglobin ^d^ Complete blood count ^e^ Protein and blood on urine dipstick ^f^ Days -t _1_, 0 and t _1_ may be the same day ^g^ Upon discharge from hospital RDT – rapid diagnostic test.

## Assessments

### HIV RDT

All participants will be offered an optional HIV rapid diagnostic test (RDT) at admission.

### Malaria RDT

A malaria RDT will be performed for all patients at admission.

### Pregnancy testing

All WOCBP (see Supplementary File 1) must take a urine/serum pregnancy test prior to enrolment.

In each case of delayed menstrual period (over one month between menstruations), the participant must take a urine/serum pregnancy test to confirm the absence of pregnancy.

### Vital signs

The following assessments will be made at least three times daily:

- Temperature
- Blood pressure
- Heart rate
- Oxygen saturation
- Level of consciousness

Assessment of urine output will be at the discretion of the treating clinician on a case-by-case basis (see also recommendations for shock and AKI in Supplementary File 2).

### Audiometry

Hearing loss will be assessed using an audiometer on admission and discharge from hospital.

### Haematology

Complete blood count, including Hb, will be conducted at least every 72 hours for the first 14 days of hospitalisation.

### Biochemistry

A blood samples will be taken for the following investigations at least every 72 hours for the first 14 days of hospitalisation:

- Serum creatinine
- Liver function
  ○ Aspartate aminotransferase (AST)
  ○ Alanine aminotransferase (ALT)

### Urinalysis

A urine dipstick test should be performed on admission.

### Lassa fever RT-PCR

Confirmation of LF will be conducted by Lassa virus RT-PCR as part of routine care before enrolment.

Subsequent blood samples for RT-PCR testing will be collected for research purposes at the timepoints specified in 5.

## Sample size {14}

**Figure 2.**
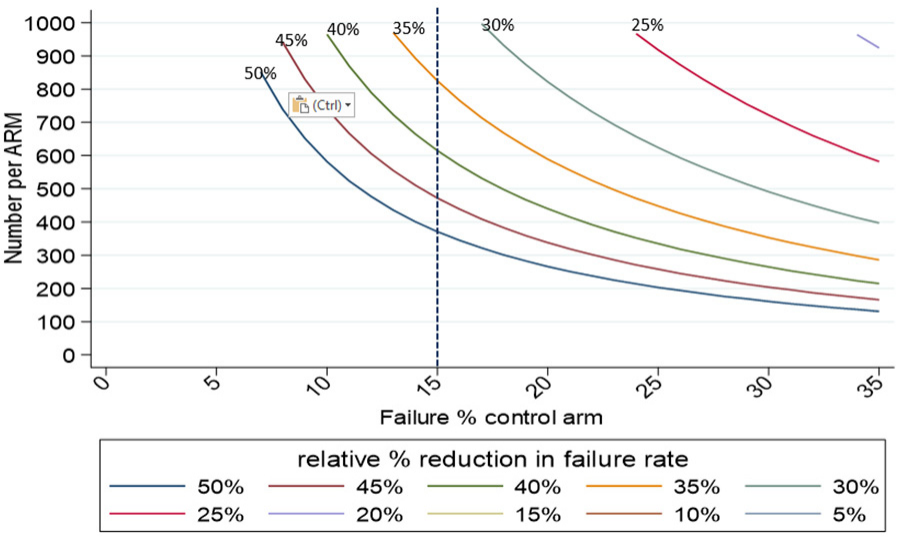
Sample size estimations for mortality endpoint.

The sample size calculation will initially conservatively assume the frequency of composite outcome is the same as the all-cause mortality outcome (13–15% mortality in the control arm based on previous studies, including unpublished data) ^20^. Table 6 and Figure 2 present sample size calculations for a range of relative risk reductions, assuming a mortality outcome of 15% in the control arm, 90% power and 10% loss to follow-up.

**Table 6.** Initial sample sizes.

Relative reduction	Sample size (per arm)
*50%*	371
*35%*	825
*≤30%*	>1000

**Suggestion:**

Please modify the previous paragraph to state the assumption on relative risk reduction that is judged relevant for the trial.

As explained above, after 300 patients are recruited or at the end of the first year of study, an analysis of the validity of the composite endpoint will be performed, and the required sample size will be re-estimated based on the data on frequency of the composite outcome. The approach to be used in the sample size re-estimation will be described in the statistical analysis plan that will be shared with the independent panel responsible for the interim analysis of validity of the composite endpoint before the initiation of the study. To ensure trial integrity ^38^, details on the sample size re-estimation and decision process will not be shared with investigators to avoid indirect inferences from the interim data. Importantly, as suggested by the FDA guideline for adaptive designs ^39^, the independent panel will need to be involved in the discussions on the details of the design and discuss potential scenarios with the sponsor in advance.

**Suggestion:**

Whilst the trial team have multiple options when considering the sample size re-estimation, below are some considerations:

(i) FDA guideline on adaptive designs suggest that non-comparative interim results have only limited effect on type I error; ^39^

(ii) Comparative approaches are also available, but involve unblinding; approaches are available to preserve type I error by combining test statistics or p-values from different stages of the trial.

If investigators decide to use efficacy stopping rules in interim analyses, stringent criteria will be used. In particular, the O’Brien-Flemming and Lan-DeMets alpha spending approaches will be considered to preserve type I error ^42^. Nonbinding futility stopping rules do not affect type I error ^39^. As the primary outcome being considered is defined within a relatively short time window, stopping rules analyses would use the same endpoint as the final analysis.

**Suggestion:**

Although there is overlap in the objectives of sample size re-estimation and group sequential design stopping rules, the trial team could be interested in having stopping rules, in addition to the sample size estimation. If this is the case, the following could be considered:

(i) For futility, several approaches are possible (conditional power, group sequential approach). Nonbinding futility rules are preferred over binding rules ^43^.

(ii) If comparative sample size re-estimation is performed, interim analyses beyond the first initial analyses might not be necessary.

(iii) Statistical software programmes for different approaches that combine sample size re-estimation and stopping rules in the interim analysis are available for example in 44.

## Recruitment {15}

The participant or their representative must personally sign and date the latest approved version of the informed consent form before any trial specific procedures are performed.

### Screening and eligibility assessment

Patients who are clinically suspected of Lassa fever will be identified and approached to participate in the trial by a member of staff at the participating site, who has been trained on the trial protocol and delegated this task by the site Investigator.

A suitably trained and qualified member of the site trial team will check that the patient meets the enrolment criteria before the patient is approached. The inclusion criteria and exclusion criteria must also be checked.

The enrolment and eligibility criteria will be cross-checked by the site Investigator (or a co-Investigator where the site Investigator completes the initial enrolment and eligibility assessment).

### Consent and assent {26a}

The participant must personally sign and date the latest approved version of the informed consent form before any trial specific procedures are performed.

Written and verbal versions of the participant information sheet and informed consent form will be presented to the participants detailing no less than: the exact nature of the trial; what it will involve for the participant; the implications and constraints of the protocol; the known side effects and any risks involved in taking part. It will be clearly stated that the participant is free to withdraw from the trial at any time for any reason without prejudice to future care, without affecting their legal rights and with no obligation to give the reason for withdrawal.

The participant will be allowed as much time as wished to consider the information, and the opportunity to question the Investigator, their medical practitioner or other independent parties to decide whether they will participate in the trial. Written informed consent will then be obtained by means of participant dated signature and dated signature of the person who presented and obtained the Informed Consent. The person who obtained the consent must be suitably qualified and experienced, and have been authorised to do so by the Chief/Principal Investigator. A copy of the signed informed consent will be given to the participant. The original signed form will be retained at the trial site.

### Adults who lack the capacity to provide informed consent

If a potential participant does not have the capacity to provide consent for themselves, a suitable consultee should be sought by the site study team. In the first instance, the site study team should try to identify a “personal consultee”, which means a person who is:

- engaged in caring for the participant (not professionally or for payment) or is interested in his/her welfare, and
- is prepared to be consulted.

This will normally be the participant’s usual carer or another person closely concerned with their welfare. This may or may not be the nearest relative.

If no appropriate person can be identified who is willing to act as a personal consultee, the researcher may consult a 'nominated consultee', i.e. a person independent of the project.

It is a matter of judgment for the researcher, in consultation with the participant’s care team, to identify the most appropriate person to act as a consultee. The responsibility to decide whether the participant should be entered into the research at all lies ultimately with the researcher.

Once a suitable consultee has been identified, they will be provided with the appropriate consultee information sheet, which includes the same level of information that the participant would receive if they had capacity.

**Note**: For those lacking capacity but with some measure of understanding, they should be provided with a simplified information sheet.

Nominated and personal consultees will complete the relevant record of consultation form which will contain their advice about the inclusion of the participant in the study.

### Adults who are unable to read

For adults who are unable to read, verbal information will be provided describing no less than: the exact nature of the trial; what it will involve for the participant; the implications and constraints of the protocol; the known side effects and any risks involved in taking part. It will be clearly stated that the participant is free to withdraw from the trial at any time for any reason without prejudice to future care, without affecting their legal rights and with no obligation to give the reason for withdrawal.

If the patient wishes to consent to participate, they will provide a finger print in place of their signature on the informed consent form.

A witness who can read must be present during the consent process to ensure the verbal information provided is coherent with that of the patient information sheet and consent form. The witness must also sign the informed consent form.

Additional documentation of this process will be in compliance with national regulations.

### Children

In the event that children should be included in later iterations of the trial, age-appropriate information will be provided to the patient explaining key elements of the trial after which the child will be given the opportunity to ask questions and assent will be requested. Active objection will be taken seriously in all children, regardless of age. The assent process will be documented in the medical notes and children will be asked to complete an assent form where they are of an appropriate age to do so.

Written consent will be obtained from a parent or caregiver following the process described above.

### Enrolment

 After the patient has signed the consent form and the enrolment and exclusion criteria have been checked by the site Investigator, the patient can be enrolled in the study and any trial-specific procedures can be carried out.

**Please note**: patients can start treatment as soon as they have been enrolled.

### Inclusion

Upon receipt of a positive RT-PCR result for LF, the patient can be included in the trial providing they (or their representative) sign the informed consent form.

## Allocation

This is a randomised trial. All patients will be randomised to treatment following consent and enrolment in the trial. This applies to patients who are enrolled before the interim analysis and after the interim analysis. 

### Sequence generation {16a}

Sequence generation will be stratified by study centre.

**Additional information required:**

The trial team needs to describe the method that will be used for random sequence generator here. See the SPIRIT statement for additional guidance.

### Concealment mechanism {16}

**Additional information required:**

The trial team needs to describe the concealment mechanism used.

### Implementation {16c}

**Additional information required:**

The trial team needs to describe the implementation of the concealment mechanism used.

## Blinding and masking {17a}

As this study is a platform trial in which new drugs with potentially diverse formulations can be added at any time, it may not be possible for either participants or healthcare staff to be blind to allocation.

The study database will be implemented so that researchers involved in the conduct and analysis of the study, who are not part of the patient’s healthcare team, can access and analyse data blind to treatment allocation.

The Data Safety and Monitoring Board (DSMB) will be the only group outside the patient’s healthcare team who may be provided with data containing treatment allocation.

### Emergency unblinding {17b}

It is unlikely that neither the participant nor their direct healthcare team will be blinded to treatment allocation. The DSMB will maintain oversight of the study with access to unblinded data on request.

## Data collection methods {18a}

All trial data will be entered on to paper case report forms (CRFs) and/or entered on to eCRF software. 

The participants will be identified by a unique trial specific number and/or code in any database. The name and any other identifying detail will NOT be included in any trial data electronic file.

No identifiable, personal data will be retained centrally (i.e. by the sponsoring organisation), but rather this will be held at individual sites only.

**Additional information required:**

Describe where, and for how long data and/or samples will be retained depending on local regulations in participating countries.

Please refer to the data management plan for further details.

### Retention {18b}

All participants who complete scheduled trial visits until D28 will have fulfilled the clinical and laboratory evaluation requirements of the trial.

Once a patient is randomized, the study site will make every reasonable effort to follow the patient for the entire study period.

Study site staff are responsible for developing and implementing local standard operating procedures to achieve this level of follow-up.

### Participant withdrawal

The type of withdrawal and reason for withdrawal will be recorded in the CRF.

During the course of the trial a participant may choose to withdraw early from the trial treatment at any time. Participants may choose to stop treatment and/or study assessments but may remain on study follow-up. This may happen for a number of reasons, including but not limited to:

1) The occurrence of what the participant perceives as an intolerable adverse event (AE).
2) Inability to comply with trial procedures
3) Participant decision.

Participants may also withdraw their consent, meaning that they wish to withdraw from the study completely. How participants wish to withdraw their consent must be recorded on the CRF:

1) Participants may withdraw from active follow-up and further communication but allow the trial team to continue to access their medical records and any relevant hospital data that is recorded as part of routine standard of care;
2) Participants can withdraw from the study but permit data and samples obtained up until the point of withdrawal to be retained for use in the study analysis. No further data or samples would be collected after withdrawal;
3) Participants can withdraw completely from the study and withdraw the data and samples collected up until the point of withdrawal. The data and samples already collected would not be used in the final study analysis, but they may have been used in any interim analyses that have taken place before the participant’s withdrawal of consent.

In addition, the Investigator may discontinue a participant from the trial treatment at any time if the Investigator considers it necessary for any reason including, but not limited to:

1) Pregnancy
2) Ineligibility (either arising during the trial or retrospectively having been overlooked at screening)
3) Significant protocol deviation
4) Significant non-compliance with treatment regimen or trial requirements
5) An adverse event which requires discontinuation of the trial medication or results in inability to continue to comply with trial procedures
6) Disease progression which requires discontinuation of the trial medication or results in inability.

Participants who withdraw from trial treatment due to the decision of the Investigator will continue to attend scheduled trial follow-up visits where possible and appropriate. Alternatively, if the reason for the participant’s withdrawal means they are unable to attend follow-up visits, data should be collected from medical records following any further visits or procedures as part of routine care.

If the participant is withdrawn due to an adverse event, the Investigator will arrange for follow-up visits or telephone calls until the adverse event has resolved or stabilised.

If a participant is withdrawn from treatment due to pregnancy the pregnancy will be followed-up to outcome. 

## Data management {19}

The data management aspects of the study are summarised here with details fully described in the data management plan. 

### Source data

Source documents are where data are first recorded, and from which participants’ CRF data are obtained. These include, but are not limited to, hospital records (from which medical history and previous and concurrent medication may be summarised into the CRF), clinical and office charts, laboratory and pharmacy records, diaries, microfiches, radiographs, and correspondence.

CRF entries will be considered source data if the CRF is the site of the original recording (e.g. there is no other written or electronic record of data). All documents will be stored safely in confidential conditions. On all trial-specific documents, other than the signed consent, the participant will be referred to by the trial participant number/code, not by name.

### Access to data

Direct access will be granted to authorised representatives from the Sponsor, host institution and the regulatory authorities to permit trial-related monitoring, audits and inspections.

## Statistical methods

### Outcomes {20a}

For the primary endpoint, the intervention arm(s) will be compared to the control arm. Unadjusted one-tailed test with significance level of 0.025 will be used; note that if efficacy or futility rules are used, interim analyses will control for type I error (e.g. using O’Brien-Flemming and Lan-DeMets alpha spending approaches). The primary endpoint to be analysed in the final analysis will depend on the findings of the initial interim analysis to validate the endpoint, i.e. a single or composite outcome measure.

Assuming the composite outcome is found to be valid, the final analysis will evaluate the primary endpoint based on the occurrence of new events, as a modified intention-to-treat analysis (mITT). Patients who meet the criteria for having an event at admission would not be eligible to experience that same component outcome in the mITT analysis; however, the occurrence of another component outcome) would be considered for the primary endpoint.

Each comparative analysis between treatment arms (interim or final) will only include concurrently randomised control data.

A statistical analysis plan will be prepared and shared with the DSMB for review and approval before the start of the trial.

**Additional information required:**

Specify each analysis the study team intends to carry out comparing study groups.

### Additional analyses {20b}

In addition to the primary comparison described above, adjusted analyses will be performed using multivariate logistic regression. Variables that will be used for adjustment include sex, age and clinical severity at presentation. Modification of the effect of the treatment on the frequency of the primary endpoint by age and clinical severity will be assessed at the multiplicative scale by including an interaction term in the regression model; effect estimates will also be presented by strata of these variables. In addition to comparisons of frequencies of the primary endpoint, the frequency of the death outcome will also be compared between treatment arms, as will secondary outcomes; the latter analyses will be unadjusted.

**Additional information required:**

Specify any additional or subgroup analyses the study team intends to carry out.

### Analysis population

**Additional information required:**

As described above, the primary analysis for each pairwise comparison will be an intention-to-treat analysis modified as described above.

## Safety reporting {22}

### Reporting period

Adverse events (AEs) and serious adverse events (SAEs) must be reported from consent until 30 days after the patient received their last dose of study treatment, unless the site investigator considers that the event is related to the study treatment in which case AEs and SAEs should be reported at any time until the end of the study.

AEs and SAEs occurring after a subject is discontinued from the study will not be reported unless the investigator determines that the event may have been caused by the study drug or a study procedure.

### Definitions of adverse events

**Table:** 

Adverse event (AE)	Any untoward medical occurrence in a participant to whom a medicinal product has been administered, including occurrences which are not necessarily caused by or related to that product.
Adverse reaction (AR)	An untoward and unintended response in a participant to an investigational medicinal product which is related to any dose administered to that participant. The phrase "response to an investigational medicinal product" means that a causal relationship between a trial medication and an AE is at least a reasonable possibility, i.e. the relationship cannot be ruled out. All cases judged by either the reporting medically qualified professional or the Sponsor as having a reasonable suspected causal relationship to the trial medication qualify as adverse reactions.
Serious adverse event (SAE)	A serious adverse event is any untoward medical occurrence that: • results in death • is life-threatening • requires inpatient hospitalisation or prolongation of existing hospitalisation • results in persistent or significant disability/incapacity • consists of a congenital anomaly or birth defect*. Other ‘important medical events’ may also be considered a serious adverse event when, based upon appropriate medical judgement, the event may jeopardise the participant and may require medical or surgical intervention to prevent one of the outcomes listed above. NOTE: The term "life-threatening" in the definition of "serious" refers to an event in which the participant was at risk of death at the time of the event; it does not refer to an event which hypothetically might have caused death if it were more severe. *NOTE: Pregnancy is not, in itself an SAE. In the event that a participant or his/her partner becomes pregnant whilst taking part in a clinical trial or during a stage where the foetus could have been exposed to the medicinal product (in the case of the active substance or one of its metabolites having a long half-life) the pregnancy should be followed up by the investigator until delivery for congenital abnormality or birth defect, at which point it would fall within the definition of “serious”.
Serious adverse reaction (SAR)	An adverse event that is both serious and, in the opinion of the reporting Investigator, believed with reasonable probability to be due to one of the trial treatments, based on the information provided.
Suspected unexpected serious adverse reaction (SUSAR)	A serious adverse reaction, the nature and severity of which is not consistent with the reference safety information for the medicinal product in question set out: • in the case of a product with a marketing authorisation, in the approved summary of product characteristics (SmPC) for that product • in the case of any other investigational medicinal product, in the approved investigator’s brochure (IB) relating to the trial in question.

### Assessment of causality

The relationship of each adverse event to the trial medication must be determined by a medically qualified individual according to the World Health Organisation – Uppsala Monitoring Centre definitions ^45^:

**Unassessable/unclassifiable:** Report suggesting an adverse reaction; Cannot be judged because information is insufficient or contradictory; Data cannot be supplemented or verified

**Conditional/unclassified**: Event or laboratory test abnormality; More data for proper assessment needed; Additional data under examination

**Unlikely**: Event or laboratory test abnormality, with a time to drug intake that makes a relationship improbable (but not impossible); Disease or other drugs provide plausible explanations

**Possibly**: Event or laboratory test abnormality, with reasonable time relationship to drug intake; Could also be explained by disease or other drugs; Information on drug withdrawal may be lacking or unclear

**Probably**: Event or laboratory test abnormality, with reasonable time relationship to drug intake; Unlikely to be attributed to disease or other drugs; Response to withdrawal clinically reasonable; Re-challenge not required

**Certain**: Event or laboratory test abnormality, with plausible time relationship to drug intake; Cannot be explained by disease or other drugs; Response to withdrawal plausible (pharmacologically, pathologically); Event definitive pharmacologically or phenomenologically (i.e. an objective and specific medical disorder or a recognised pharmacological phenomenon); Re-challenge satisfactory, if necessary.

Events that are considered possibly, probably and certainly related to the trial drugs will be classified as ((suspected unexpected) serious) adverse reactions. Events that are unassessable/unclassifiable, conditional/unclassified and unlikely to be related to the trial drugs will be classified as serious adverse events only.

### Expectedness

Expectedness of SARs will be determined according to the relevant RSI section of the Investigators’ brochure/summary of product characteristics. The RSI used will be the current Sponsor approved version at the time of the event occurrence.

### Procedures for reporting adverse events

All AEs occurring during the safety window for the trial as defined above that are observed by the Investigator or reported by the participant, will be reported on the trial CRF.

The following information will be reported on the CRF: description, date of onset and end date, severity, assessment of relatedness to trial medication, other suspect drug or device and action taken. Follow-up information should be provided as necessary.

The severity of events will be assessed on the following scale: 1 = mild, 2 = moderate, 3 = severe, 4 = life-threatening, 5 = fatal.

All non-serious AEs will be followed up until resolution.

It will be left to the treating clinician’s clinical judgment to decide whether or not an AE is of sufficient severity to require the participant’s removal from the trial. A participant may also voluntarily withdraw from treatment due to what he or she perceives as an intolerable AE. 

### Reporting procedures for serious adverse events

All SAEs (other than those defined in Section 17.6 as not requiring reporting) must be reported on the SAE reporting form to the Sponsor or delegate within 24 hours of site study team becoming aware of the event being defined as serious.

The site study team will complete an SAE report form for all reportable SAEs with as much information as is available at the time of reporting.

The SAE report form will be scanned and emailed to the Sponsor contact/entered on to the SAE eCRF within 24 hours of site study team becoming aware of the event.

The site study team will provide additional, missing or follow up information in as soon as it becomes available.

The Sponsor will provide a causality assessment of the SAE within 1 business day of receipt.

If the SAE is a SAR, the Sponsor will perform an expectedness assessment using the Sponsor-approved Reference Safety Information (RSI) current at the time of the event.

### Events exempt from immediate reporting as SAEs

The following events do not require reporting as SAEs:

- Hospitalisation for a pre-existing condition, including elective procedures planned prior to study entry, which has not worsened.

**Additional information required:**

Add any other trial-specific events that may be exempt from reporting.

## Data monitoring

### Formal committee {21a}

A data and safety monitoring board (DSMB) will oversee patient safety, monitor trial conduct, and, when appropriate, assess interim data.

The DSMB will be formed of members independent of the Sponsor and study. Members will declare any competing interests ahead of being formally appointed.

The DSMB will advise the Trial Steering Committee of any actions it deems necessary for the continuation, or termination, of the study. Furthermore, an independent panel will be responsible to assess the validity of the composite endpoint and make decisions regarding sample size re-estimation.

### Interim analyses {21b}

An essential component of the design is to first characterise the frequency and validity of the proposed primary composite endpoint. After recruitment of 300 patients or after one year after recruitment, whichever happens first, an analysis of the number and percentage of patients experiencing each of the composite endpoint events at admission and within a short pre-specified time period will be performed. Specifically, the criteria that will be used by the independent panel to assess the validity of the composite endpoint consist of:

- The proportion of patients enrolled in the trial who present with one or more of the components of the composite endpoint on admission
- The proportion of patients experiencing a new event, defined as one of the components of the composite outcome, following admission
- The variation of the frequency of the events by study site and over time, as the profile of patients recruited to the trial and the time from infection to hospital admission might change.

In particular, the independent panel will assess whether the onset of any of the component events of the composite outcome typically occurs before randomisation, in which case the panel might chose to remove the component event from the definition of the composite outcome. Any challenges for the use of the composite outcome as a primary endpoint for the trial would be examined carefully.

Access to data and results would be limited to a pre-specified small group to allow an independent panel of experts, including an independent statistician, to make recommendations for decision making to approve the composite outcome as the primary endpoint of the trial. 

Only after assessment of the validity of the composite outcome, the independent panel will proceed with the following steps: (i) assessment of efficacy and/or futility stopping rules, if these were planned and described in the statistical analysis plan; (ii) sample size re-estimation.

The sample size assumptions for a trial powered to evaluate the composite endpoint as the primary endpoint would be re-estimated, based on these data and a decision would be made as to whether to make the sample size adjustment in a protocol amendment.

### Additional interim analyses

**Suggestion:**

Please specify the number and timing of additional interim analyses, and whether efficacy or futility will be tested. If no additional interim analyses are performed, this subsection can be deleted.

In addition to the analysis that will be used for the validation of the composite outcome, additional interim analyses will be performed applying efficacy (or futility) stopping rules. In the statistical analysis plan that will be shared with the DSMB before the first interim analysis, the statistical approaches taken to control for type I error and to account for potential reduction in study power will be described based on this number of interim analyses. In particular, we will prioritise use of well-established approaches, such as O’Brien-Flemming or Pocock, or alpha spending function, for control of type I error, when efficacy stopping rules are used. For futility stopping rules, which do not affect type I error, but can affect the study power ^33, 46^, group sequential approaches, including beta-spending function, or conditional power approaches can be used; recommendations on the conditional power thresholds to be used are described for example in 43.

## Harms {22}

Outcome measures related to harms are assessed as part of the primary and secondary outcome measures.

Pregnancy reporting will also be a requirement for participants who enter the study and receive a positive pregnancy test result either before or during treatment. Pregnancy reporting will also be required for partners of male participants who receive a positive pregnancy test result either before or during the participant’s treatment. In these scenarios the pregnancy will be followed up until its outcome, which should be recorded on the pregnancy report form.

## Monitoring {25}

**Additional information required:**

Please describe the monitoring procedures for the trial.

## Protocol amendments

Any amendments that are made to the protocol and associated study documents will be reviewed by the applicable ethics committees and/or regulatory authorities in the responsible countries involved in the research.

## Confidentiality {27}

All paper-based study-related information will be stored securely at the study site and all electronic study data will be held securely on servers located at the research team’s institution. Any electronic data held locally on-site will be stored on encrypted devices that are password protected with restricted access to only those authorised to work on the trial.

All study samples and data will be identified by a study participant ID only to maintain participant confidentiality. All records that contain participant names or contact information, such as informed consent forms, will be stored separately from study records identified by code number.

All study data will be protected by local laws and regulations governing data protection.

Participants’ study data will not be viewed by anyone outside the study team, except for monitoring and auditing purposes by the Sponsor organisation or regulatory bodies to review study conduct.

## Ancillary and post-trial care {30}

**Additional information required:**

Please describe the arrangements for ancillary and post-trial care here.

## Dissemination policy {31a}

**Additional information required:**

Please describe the dissemination policy here.

## Ethics approval and consent to participate {32}

This is a pre-positioned protocol. At the time of publication of this pre-positioned protocol, no study is planned. It has been written and published in preparation for adaptation and implementation by the Lassa fever research community. Any research group who implements this protocol would be required to obtain regulatory and/or ethical approval in line with local and international guidelines.

## Consent for publication {32}

Not applicable.

## Discussion

This article describes a pre-positioned protocol for the evaluation of multiple therapeutics for Lassa fever.

While it is clear that the current standard of care – ribavirin – requires reassessment due to important safety concerns, it is unlikely that a trial could be implemented without ribavirin or any other active treatment. A survey conducted by the WALC found that West African clinicians have clearly expressed their opposition to trials not including ribavirin or a putative active drug on top of supportive care, i.e. placebo-controlled trials. Further, conducting trials comparing the different ribavirin regimens is unlikely to be cost-effective and time-efficient given the additional pre-clinical and phase II pharmacokinetic studies that would be needed to do this in a robust manner. Finally, given the possible safety concerns that have been raised about ribavirin and the fact that the pre-clinical data is not comprehensive enough, it could be considered unethical to conduct any trials of ribavirin in Lassa fever.

A potential limitation of this design is that any trial of new therapy will need to be tested against ribavirin, despite ribavirin potentially being ineffective or indeed harmful. This could theoretically lead to a situation in which a new drug is found to be superior to ribavirin, when in fact it represents the new drug simply not being harmful in comparison to ribavirin. A potential solution to this problem is to replace ribavirin with the new drug in the control arm if the efficacy of a new drug is established.

The safety and efficacy of new treatments assessed under this protocol will be evaluated using a composite primary endpoint that consists of mortality or new onset of three critical Lassa fever complications: Acute Kidney Failure, Acute Respiratory Failure or shock. It is clear that, as death occurs in approximately 12% of Lassa fever cases managed in a hospital setting – a relatively low frequency to detect significant improvements with new treatments – using mortality alone as a primary outcome measure in a clinical trial would generate an unfeasibly large, unachievable sample size.

The challenge in implementing this composite primary outcome measure is that insufficient information exists on the frequencies of the above complications which hinders the accurate estimation of the trial’s sample size. To obtain an accurate estimate of the required sample size, the trial would be initially powered for a mortality outcome measure, but an interim analysis would be conducted after 300 patients have been included in the study to assess the frequencies of the events in the composite outcome measure and validate the use of a composite outcome measure. These data would then be used to confirm the required sample size to provide adequate power for subsequent analyses.

This pre-positioned protocol was developed by the WALC and made available for adaptation and implementation by the wider Lassa fever research community in order to generate efficient, reliable, and comparable evidence for Lassa fever therapeutics. This protocol has been reviewed by clinicians, regulators, researchers and members of ethics committees in West Africa and other international stakeholders.

## Abbreviations

AE – Adverse Event

AKF – Acute Kidney Failure

AKI – Acute Kidney Injury

ALT – Alanine Aminotransferase

ARF – Acute Respiratory Failure

AST – Aspartate Transferase

CRF – Case Report Form

CTCAE – Common Terminology Criteria for Adverse Events

D – Study day

DSMB – Data Safety and Monitoring Board

FDA – United States Food and Drug Administration

Hb – Haemoglobin

ITT – Intent to Treat analysis

KDIGO – Kidney Disease Improving Global Outcomes

LBW – Low Birth Weight

LF – Lassa fever

MAMS – Multi-arm Multi Stage design

MAP – Mean Arterial Pressure

mITT – Modified Intent to Treat analysis

PD – Pharmacodynamics

PK – Pharmacokinetics

RDT – Rapid Diagnostic Test

RSI – Reference Safety Information

RT-PCR – Reverse Transcription Polymerase Chain Reaction

SAE – Serious Adverse Event

SAR – Serious Adverse Reaction

SGA – Small for Gestational Age

WALC – West Africa Lassa fever Consortium

WHO – World Health Organisation

WOCBP – Women of Childbearing Potential

## Data Availability

No underlying data are associated with this article.

## Reporting guidelines

This article has been written according to the SPIRIT guidelines.
