# Supplementary material for: The West Africa Lassa fever Consortium pre-positioned protocol for a Phase II/III adaptive, randomised, controlled, platform trial to evaluate multiple Lassa fever therapeutics
Source: Wellcome Open Res. 2023 Jun 2;8:122. Originally published 2023 Mar 16. [Version 2] doi: 10.12688/wellcomeopenres.19041.2 (PMC11358687; doi:10.12688/wellcomeopenres.19041.2)
Supplement: Peer review history

# Reviewer report: Reviewer response for version 2

**DOI:** 10.21956/wellcomeopenres.21622.r93826

**Published:** 2024-09-05

**Authors:** Juan de la Torre

**Affiliations:** 1 The Scripps Research Institute, La Jolla, California, USA

This is an updated version of paper proposing the structure of a standardized, pre-positioned protocol for a Phase II/III clinical trial to evaluate multiple therapeutics for the treatment of Lassa fever disease caused by the mammarenavirus Lassa virus (LASV).

 This updated version 2 of the paper has incorporated the appropriate changes to address the issues raised by Dr. George O. Akpede about the originally submitted paper.

 I concur with Dr.Emmanuel Bottieau that this updated version still lacks details about the specific regimen for the ribavirin treatment that will be used as benchmark. 

 In addition, I think that the protocol should provide a more detailed description about the RT-PCR procedures, as the readout of this assay will be used as a main inclusion criteria. Details about the protocols and controls to be used across the different sites should be established ahead of initiating recruitment. It would be important to establish experimental conditions to minimize the possibility of false negatives due to lack of genetic coverage.

Is the study design appropriate for the research question?

Yes

Is the rationale for, and objectives of, the study clearly described?

Yes

Are sufficient details of the methods provided to allow replication by others?

Yes

Are the datasets clearly presented in a useable and accessible format?

Not applicable

Reviewer Expertise:

My area of expertise is molecular and cell biology of mammarenaviruses including LASV, and development of therapeutics against hemorrhagic fever causing mammarenaviruses.

I confirm that I have read this submission and believe that I have an appropriate level of expertise to confirm that it is of an acceptable scientific standard.

# Reviewer report: Reviewer response for version 2

**DOI:** 10.21956/wellcomeopenres.21622.r77058

**Published:** 2024-08-28

**Authors:** David Simons

**Affiliations:** 1 Department of Anthropology, The Pennsylvania State University, State College, PA, USA

Pre-positioned protocols for platform trials for sporadic or outbreak associated endemic zoonoses are an excellent opportunity to allow healthcare researchers to rapidly respond to outbreaks and guide the production of an evidence base for safety and efficacy of novel or repurposed drugs in clinical management. The development and implementation of the RECOVERY trial and PANORAMIC trial in COVID-19 was vital in providing a secure evidence base for COVID-19 treatments and the development of a similar approach for Lassa fever would be fantastic.

 This protocol begins this process for Lassa fever and has been developed by the West African Lassa Fever Consortium. The consortium comprises collaborators and centers in the Lassa fever endemic region with direct experience in the identification of individuals suffering with symptomatic Lassa fever and their management.

 As the WALC highlight clinical trials are urgently needed for the management of Lassa fever in endemic settings. Developing and making this protocol available is a vital first step in improving the quality of clinical evidence for unproven therapeutics. I would hope that consortia such as the WALC that encompass specialist clinical settings across the endemic region are able to action this protocol with appropriate support in the near future.

 I have no major comments. The protocol appears well thought through with clear directions to those who will implement it what further information would be required.

 Minor comments.

 My understanding of the patient pathway for LF, in Sierra Leone at least, is that patients are referred to the specialist centre rather than admitted from within the site. This may limit the approach for screening and enrolment if the recruitment team is solely based at the tertiary centre. It may be worth considering the need for satellite recruitment to reduce the delay in enrolment if current processes are followed.

 "Transmission to humans occurs primarily through contact with contaminated surfaces" - Perhaps consider changing to "Transmission to humans is expected to occur primarily....". The evidence for mode of infection in Lassa fever is unclear and to my knowledge it is unclear whether direct contact with contaminated surfaces or aerosolisation of rodent excreta lead to human infection.

 In the paragraph describing the symptomatology of LF it may be worth highlighting that a substantial number of infections are likely asymptomatic, given the incongruence between human serology studies and the identification of clinical cases in endemic regions. This could be considered alongside the inclusion criteria for any clinical trials as asymptomatic/ pauci-symptomatic LF may not be suitable for consideration for enrolment and treatment given concerns about side-effect rates in current Standard-of-care treatment with Ribavirin. It may be that this represents a very small proportion of individuals that are confirmed to be infected with LF but should asymptomatic infections be excluded from enrolment?

 The justification for the potential use of the composite endpoint and the associated sample size estimations are clear and appear valid.

 Choice of comparators. The issues around evidence for Ribavarin have previously been discussed. It would be of interest to know how prevalent it's use is currently. Is there available information on how many of the hospitalised cases within the WALC receive Ribavirin and how many don't?

 Respiratory failure. No changes required for this protocol but it may be worth considering the accuracy of SpO2 monitoring in endemic settings (i.e., Henry NR, et. al., 2022 [Ref 1])

 Is it intended that consent forms will be provided in English or the participants preferred language?

 Date of symptom onset may be a valuable assessment to aid interpretation as part of the additional analysis given expected delays in presentation.

Is the study design appropriate for the research question?

Yes

Is the rationale for, and objectives of, the study clearly described?

Yes

Are sufficient details of the methods provided to allow replication by others?

Yes

Are the datasets clearly presented in a useable and accessible format?

Not applicable

Reviewer Expertise:

Medical doctor (unspecialised), epidemiology and rodent associated disease ecology.

I confirm that I have read this submission and believe that I have an appropriate level of expertise to confirm that it is of an acceptable scientific standard.

## References

[1] : Disparities in Hypoxemia Detection by Pulse Oximetry Across Self-Identified Racial Groups and Associations With Clinical Outcomes. *Crit Care Med* .2022;50(2) : 10.1097/CCM.0000000000005394 204-211 10.1097/CCM.0000000000005394 35100193 PMC9070439

# Reviewer report: Reviewer response for version 2

**DOI:** 10.21956/wellcomeopenres.21622.r59181

**Published:** 2023-06-21

**Authors:** Emmanuel Bottieau

**Affiliations:** 1 Department of Clinical Sciences, Institute of Tropical Medicine, Antwerp, Belgium

Thank you for all clarifications regarding this great protocol, which is now fine to me. As a final comment, I still regret however that there is no more available information on:

1. The current knowledge on the disease characterization:
  I understand that it is challenging to provide a synopsis of the Lassa complications, at presentation and during the course of the disease, because the data are extremely heterogeneous. This issue has been debated by the WALC group apparently, and the information was perceived as “insufficient” to establish an adequate composite primary endpoint. It is a pity that no meeting minutes are provided in a transparent way to support this conclusion. Most external readers and researchers are used to these kinds of study limitations. The collected information could have been summarized with some warnings, and prioritizing the studies of highest quality (prospective, large sample size,…). This knowledge gap leads to the innovative - but unusual - two-stage design of this trial. Classically, good prospective observational studies on a disease are the necessary step prior to the design and conduct of interventional trials. I hope this approach “under constrains” will be understood and accepted by trialists and ethicists.
2. The choice of the ribavirin regimen(s) for the trial:
  For the same reason, there seems to exist no consensus within the WALC group on the best ribavirin regimen to offer in the control arm of the trial. Here again providing a Table summarizing the pros and cons of the different regimens “in competition” would be great, as this is a step any researcher will need to undertake while designing the trial. If the rationale to select an option is also considered as insufficient, the preference should likely go the safest regimen.   

I suggest these two limitations related to the lack of consensus between experts are more clearly stated in the Discussion and mentioned also in the amendment, as major points of attention for the further trial development.

Is the study design appropriate for the research question?

Yes

Is the rationale for, and objectives of, the study clearly described?

Partly

Are sufficient details of the methods provided to allow replication by others?

Partly

Are the datasets clearly presented in a useable and accessible format?

Not applicable

Reviewer Expertise:

Medical doctor, specialized in Internal Medicine, Infectious and Tropical Diseases (clinician)

I confirm that I have read this submission and believe that I have an appropriate level of expertise to confirm that it is of an acceptable scientific standard.

# Reviewer report: Reviewer response for version 1

**DOI:** 10.21956/wellcomeopenres.21110.r55702

**Published:** 2023-05-04

**Authors:** George O. Akpede

**Affiliations:** 1 Irrua Specialist Teaching Hospital, Irrua, Nigeria

**Introductory remarks:**

 There is no doubt that a new effective product for the treatment of Lassa fever, a long-neglected tropical disease, could have more than a salutary impact on the outcome but its efficacy would need to be shown in clinical trials. Bourner *et al.*’s work ^1^ in developing a de facto master protocol for phases II/III trials of such products is thus to be highly welcomed. The authors have carried out an important assignment with due diligence, thoughtfulness and foresight and should be commended. Nonetheless, it seems to me also important to acknowledge and realistically appraise the place of ribavirin in the treatment of LF, not minding the recent controversies as to its efficacy and safety in patients with LF. Added to these two issues, which are interrelated, is the third issue of what should be the totality of the expected benefits from the forthcoming clinical trials of LF vaccines and new therapeutics. My comments and remarks on the protocol are modulated by these three considerations. Before I proceed, however, I should note that the paper is generally well written, and indeed, I was tempted to recommend it outright for approval but for a few observations that might further strengthen the message(s) therein.

 For ease of understanding but perhaps also of convenience and considering their potential significance, I have elected to present my remarks in three groups: First, observations and remarks of a cross-cutting nature that in my view could have impact for the conceptualization, rationale and design of the protocol; second, issues that might have to do with writing style but does have potential for impact on protocol implementation and clinical outcomes. These might therefore also have ethical connotations; and third, issues which I think should be clarified to enhance understanding and facilitate implementation of the protocol. The three introductory remarks which I made in the preceding paragraph have in the main to do with the cross-cutting issues. At the same time, however, I should emphasize that neither of the group of comments is of course, expected to be unconnected.

**Observations and remarks of a cross-cutting nature with potential for impact on the conceptualization, rationale, and design of the protocol:**

 The paper ^1^ has some fundamental uncertainties and contestable claims, that could have been resolved or avoided through enhancement of the background information from a more comprehensive review of the available pool of observational data. I did not find any or sufficient reference to this in the paper and consider it a major weakness, with its implications for the rationale undergirding the recommendation for platform trials, study objectives, protocol design and recommendations on choice of endpoints:

 It may be necessary to revisit the **justification for the choice of platform trials and study design**. For example, a more comprehensive review of extant literature, especially from recent times, ^2-7^ might not support the claim that cases of LF in endemic areas are “few and sporadic” with a “small number of patients” excepting if these descriptions are made to take on meanings beyond their ordinary usage. Then, the allusion made to some parallels between LF, and Ebola may have overlooked important differences between them that might be of equal or greater significance. For example, LF outbreaks ^2,3,7-10^ are typical annual and recurrent unlike Ebola outbreaks ^11^ and whereas until recently there was no ‘specific treatment’ for Ebola, ribavirin, formally approved or not, has been available for decades for the treatment of LF ^12^ not minding the recent controversies as to its safety and efficacy. ^13,14^ And, granted that there are a “small number of trial sites … in Lassa-endemic areas” could the number not be built up before the proposed trials in order that it does not become a permanent fixture? And with respect to the worry that “the customary approach of individual separate trials” could be wasteful, we don’t think that this line of argument is sustainable.

 Then, the position taken on ribavirin seems too conclusive when weighed against the available evidence. I suggest that it be reviewed as it has implications for the trial protocol design. The recent reviewers of the McCormick *et al.*’s ^12^ trial report and data did not discount the effectiveness of ribavirin in the treatment of severe LF and other than the concerns expressed over the study design and execution, the major reservation were questions of the efficacy and safety concerns among putatively mildly ill patients (defined as those with AST levels <150 iu/L) treated with ribavirin. ^13,14^  Bourner *et al.* ^1^ should have interrogated some of the conclusions from these reviews in taking a position. For example, what is the correlation between AST levels and other indices of illness severity, and could its sole use not potentially misclassify some cases? Also, to what extent have the safety concerns been confirmed in subsequent observational studies? ^4,15-17^ I do not dismiss the supposed reticence of “local clinicians” towards further trials of the efficacy of ribavirin as alluded to in the paper, ^1^ but I am of the view that this could be managed through further engagement with them. And by the way, more information should have been given on the “consultation” with them, for example, what was the nature, exactly what issues were discussed and how representative of the group were the respondents?

 Furthermore, what has the trialing the efficacy of ribavirin got to do with “conducting trials comparing the different ribavirin regimens” and for that matter on what basis was it thought that such a trial “is unlikely to be cost-effective”? In the end, no ribavirin regimen was recommended for use in the protocol but is it not intuitive that the ‘Irrua regimen’ of a single daily dose ^18^ could be cost-saving given the reductions in caregiver-patient contact time, and in material and drug requirements? And which practice might really be “unethical”, continuation with the use on a large scale of a drug with such potentially serious safety concerns or conducting further investigations to resolve the controversy? The opposite view that there are important reasons for an urgent trial of ribavirin (versus placebo versus new product) in the treatment of mild LF is in my view not unreasonable as it would, likely, remain in use in endemic areas for now.

 In addition, further thought should be given to the recommended use of ribavirin as comparator in the trials. As it were, the recent ribavirin controversy ^13,14^ has to do with its place in the treatment of mild ill patients whereas all agree as to its effectiveness in the severely ill. ^12-14^ Its choice as a comparator drug may therefore be appropriate only for trials among severely patients. Why not recommend all potential therapeutic agents including ribavirin for placebo-controlled trials in mildly ill patients?

 Finally, given that only ribavirin has been available for decades ^12^ and now favipiravir is gaining in advocacy for use as a repurposed alternative and/or complement, ^19^ trialing a few drugs at a time, even if individually, could be positively less disadvantageous than a platform trial that may have to wait until the “three experimental drug candidates” are ready for the trial phases envisioned. And given the demand for ribavirin and the potential benefits of the favipiravir-ribavirin combination ^19^ while being mindful of the safety concerns in treating the ‘mildly ill’ with ribavirin, why should trial of the 2 drugs have to wait for the “experimental three” and ‘potential others’ to be made ready in the face of the perceived urgency painted in the paper? ^1^ Accordingly, I wish to urge a rethink of the issues and the choice of platform trials. The ‘rationale’ for the study as presented is somewhat busy but could be simplified emphasizing the dual need of therapeutic alternatives to ribavirin plus the need for its further trialing in the treatment of mild cases of LF. It may well be then that a modified or limited platform trial of favipiravir or ‘product X’ versus ribavirin plus ‘product X’ in severe LF and of ribavirin and/or ‘product X’ versus placebo in mild cases of LF pending the availability of other therapeutic products is what we need, at least for now.

 It seems also important that the authors revisit **the choice of endpoints** for several reasons. First, there really should be no “uncertainty around the frequencies of the events included in the composite endpoint” nor as to the timing as it ought to be possible to determine these from available reports. ^4,7,15-17,20-22^ In reality, we also have more than “only very limited data” on the admission status of cases of LF. ^1^ With respect to all three issues, recent reports ^4,15,16,22^ are in harmony that complications and death are most frequent within 48-72 hours of admission, a period when the adequacy of supportive care rather than the putative efficacy of an antiviral agent may be the main determinant of outcome. ^7^ This could mean that the choice of mortality as a primary endpoint or outcome measure may underestimate the impact of pharmaceuticals and that effective supportive care might result in a dissociation between the frequencies of death and the composite outcome. In effect, the scenario in which the frequency of the composite outcome is likely to be higher than “the frequency of death outcome in the study population” should be envisaged. It seems necessary therefore that the authors revisit the issues of determination of endpoint and sample size estimation. And, by the way, why not consider using the ‘traditional’ indices of response to antimicrobial therapy such as temperature trend and time to defervescence, viral kinetics and other improvements in clinical status?

 Second, whilst the point made about the variation in availability of medical resources for supportive care ^1^ is true, this is as far as I could agree because: 1) I believe that I have already emphasized the need for and potential impact of adequate supportive care as well as addressed the purported dearth of relevant data on the frequencies of complications on admission as in the preceding paragraph. 2) The appearance of “new complications” of the types under reference ^1^ is not common; it is even also possible that the presence of such complications might have antedated admission but that the astuteness of the attending physician and/or limitations of investigational resource had constrained their identification earlier. And 3), this is fundamental. Given that the underdevelopment of resources and capacities for supportive care is a major factor in the high mortality rates in endemic areas, ^4,7,16^ should we not take firm hold of the opportunities in the preparation for the forthcoming clinical trials of vaccines and therapeutics to remedy the situation? I think that we should, indeed, as an ethical imperative that should neither be overlooked nor missed given the impact of supportive care on outcomes. ^4,7,16,23-27^ However, none of the rebuttals could, of course, mean that it is wrong to want to assess the validity and frequency of a composite outcome, or to desire to enhance the quality and amount of data, but the point is that such processes should antedate the trials.

 Regarding the other issues raised over endpoint recommendations, it is probably good to anticipate “variation of the frequency of the events by study site”, ^1^ but it should also be possible to factor this into the study design. Whilst the onset of “new” complications during admission might also be attributable to treatment failure rather than to the natural course severe LF, it seems necessary to set a minimum duration of time from admission for such occurrences although this might have to be arbitrary.

 Several issues were also highlighted in the effort to justify the choice of a composite rather than single endpoint but some of them are difficult to countenance. As examples, the claims of a low case incidence and mortality are not backed by available reports. ^2,3-7,15-17,21,22,28,29^ Take this against the background that LF may be expanding into new areas in West Africa, ^2,30^ and there may be ongoing increases in caseload and case fatality in some endemic areas. ^2,29,31^ And of course, the claim of low mortality is likely incorrect regarding severe infections ^4,7,16,21^ but even if it is so, the observation could also be factored into the design of the study.

 Finally, let us review the **rationale for the recommended objectives: **The main objective, “to develop and agree upon a methodology for the evaluation of Lassa fever therapeutics” is clear as is the “complementary objective” “to address capacity strengthening for clinical trials”, but why bring in the issues of drug development as other “complementary objectives” ^1^ which seem extraneous to the subject matter? Instead, it seems to me that the need to strengthen clinical case management capacity, in particular the capacity for supportive treatment as highlighted earlier ( **vide supra**), may flow more readily with the strengthening of clinical trial capacity, and is more practical and pragmatic. That this “requires significant investment” should not at all negate the desire. Indeed, it would be curious if inclusive capacity building is not the benchmark imperative in the run up to the trials.

 The authors seemed to have overlooked having to provide background information on what is known or uncertain about the course of LF among hospitalized patients. Is this omission also because there is a dearth of data? Its inclusion would been helpful in appraising the case made for the secondary objective “to evaluate the prevalence of complications associated with Lassa fever”. Several “other study objectives” are also listed, some in the text and some in a box but what they really have to do with the protocol should be clarified please.

**Avoidable sources of variations in protocol implementation which may also have ethical implications:**

 The protocol contains several statements that could generate variations in quality of care and outcomes between trial sites and might thus have ethical connotations which are otherwise avoidable. These include such statements as: “it may not be feasible to standardise the supportive care available to all patients enrolled across participating sites”, “All other supportive care will be at the discretion of the treating clinician”, “the decision to start dialysis will be left at the discretion of the treating clinician”, “Assessment of urine output will be at the discretion of the treating clinician on a case-by-case basis“, prescribing “oxygen support through any available method”. There is also the statement that “Steroids are not routinely recommended in patients with Lassa fever who are hypotensive”, but no clarification is made as to when steroids should be administered. Furthermore, why vaguely and unconventionally define an adult as “a person who has attained the age of majority according to national regulations in their country of enrolment“? Such perceived lack of uniformity between sites might also have the unintended but real effect of attracting patients preferentially to some. These statements should be revised if possible.

 The paper ^1^ also includes statements that may not have a meaningful level of evidence backing such as “Patients in shock should receive broad-spectrum antibiotics”, “Broad-spectrum antibiotics (ceftriaxone) should be given to all patients with signs of meningism, focal neurology or seizures” and “This protocol recommends that a blood transfusion should be provided to patients with Hb <8 g/dL”. The basis of such recommendations should be explained.

 On the other hand, beyond the recommendations of diazepam as “first line treatment for seizures” and broad-spectrum antibiotics as stated above, the protocol is silent on the need for at least a minimal level of assessment of patients with encephalopathy. Why is this so despite what is known of its prognostic significance in LF? ^4,32^

**Miscellaneous issues:**

 Clarification is required regarding several issues:

1. Beyond stating on page 3 that “The protocol is the product of discussions … to develop and agree upon a methodology for the evaluation of Lassa fever therapeutics … trials”, the authors give only very little information on the method used to derive the protocol.  The need for reproducibility warrants that at least a synopsis of the discussions including information on the research questions discussed, how consensus was achieved and disagreements resolved, and the representativeness of the discussants, amongst others, should be added under ‘Methods’.
2. Why the recourse to “Unadjusted one-tailed test with significance level of 0.025” (Statistical methods, page 15)?
3. What informed the choice of “Acute Kidney Failure, Acute Respiratory Failure or shock”, or the exclusion of bleeding and encephalopathy, as “new set of critical complications” for inclusion in ”a composite primary endpoint” (page 19, paragraph 4 of ‘Discussion’)?
4. Why set the interval between the initial blood sample RT-PCR and the next 13 days apart (Table 5)?
5. Some of the co-authors are also featured under ‘Acknowledgements’ whilst none of those alluded to in a statement in the main text of the paper ^1^ that, “This protocol has been reviewed by clinicians, regulators, researchers and members of ethics committees in West Africa and other international stakeholders” was at all listed?
6. What is the basis for setting an interval of 4 hours for the confirmation of complications?
7. What is the real difference between “Suggestion” and “Additional information required”, and is it not possible to incorporate such statements in the text? Coming up in many sections of the paper, ^1^ they give the impression of ‘a work in progress’.
8. Are the readers and users of the protocol to be left to conjecture why children were recommended for exclusion from the trials given that they currently constitute an important segment of cases, about 20-30% of hospitalized cases in some countries?
9. The assessment of clinical severity on presentation is not described anywhere in the protocol but clinical severity is listed as one of the variables for adjustment on page 16 under ‘Additional analyses”.
10. Would it be too much to expect necessary documentation to back up the claim that support for the protocol that has been obtained from regulators and West African ethics committees? 
11. And, could those adults who are not able to read not have the translations of the informed consent form read aloud to them and the whole process documented audio-visually?

The authors should also consider that the paper is somewhat long, and a reader could lose focus going through. Is it not possible to moderate its length, for example, by presenting some of the materials as supplements or by referring the reader to the original source(s) of information such as the KDIGO criteria? The latter and the listing of “Acceptable contraceptive measures” are examples of portions that could be handled this way?

 Finally, I think it is important that members of those religious organizations such as Jehovah’s Witnesses who object to blood transfusion should also be considered for exclusion from the trials. The members may not be sparse in LF-endemic areas. And I venture to whisper that reference #17 in the list seems incomplete.

**In conclusion**, I desire to restate that this protocol is important and should serve well as a guide in the forthcoming trials of new LF therapeutics, but it seems to me to be work in progress. I think strongly that a more robust appraisal of the extant literature and data could assist in the resolution of some or most of the apparent uncertainties in the protocol and indicate clearly hopefully, the need for revisions of important section including the rationale, design, and objectives.

Is the study design appropriate for the research question?

Partly

Is the rationale for, and objectives of, the study clearly described?

Partly

Are sufficient details of the methods provided to allow replication by others?

Partly

Are the datasets clearly presented in a useable and accessible format?

Not applicable

Reviewer Expertise:

NA

I confirm that I have read this submission and believe that I have an appropriate level of expertise to confirm that it is of an acceptable scientific standard, however I have significant reservations, as outlined above.

## References

[1] : The West Africa Lassa fever Consortium pre-positioned protocol for a Phase II/III adaptive, randomised, controlled, platform trial to evaluate multiple Lassa fever therapeutics. *Wellcome Open Research* .2023;8: 10.12688/wellcomeopenres.19041.1 10.12688/wellcomeopenres.19041.1 PMC1135868739211525

[2] : 52 Years of Lassa Fever Outbreaks in Nigeria, 1969-2020: An Epidemiologic Analysis of the Temporal and Spatial Trends. *Am J Trop Med Hyg* .2021;105(4) : 10.4269/ajtmh.20-1160 974-985 10.4269/ajtmh.20-1160 34460421 PMC8592130

[3] : Lassa Fever Outbreak Situation Reports. *NCDC* .2023; Reference source

[4] : Clinical and laboratory predictors of Lassa fever outcome in a dedicated treatment facility in Nigeria: a retrospective, observational cohort study. *The Lancet Infectious Diseases* .2018;18(6) : 10.1016/S1473-3099(18)30121-X 684-695 10.1016/S1473-3099(18)30121-X 29523497 PMC5984133

[5] : Genomic Analysis of Lassa Virus during an Increase in Cases in Nigeria in 2018. *N Engl J Med* .2018;379(18) : 10.1056/NEJMoa1804498 1745-1753 10.1056/NEJMoa1804498 30332564 PMC6181183

[6] : Increase in Lassa Fever Cases in Nigeria, January-March 2018. *Emerg Infect Dis* .2019;25(5) : 10.3201/eid2505.181247 1026-1027 10.3201/eid2505.181247 30807268 PMC6478197

[7] : Caseload and Case Fatality of Lassa Fever in Nigeria, 2001-2018: A Specialist Center's Experience and Its Implications. *Front Public Health* .2019;7: 10.3389/fpubh.2019.00170 170 10.3389/fpubh.2019.00170 31294014 PMC6603170

[8] : Lassa Fever: Epidemiology, Clinical Features, Diagnosis, Management and Prevention. *Infect Dis Clin North Am* .2019;33(4) : 10.1016/j.idc.2019.08.002 933-951 10.1016/j.idc.2019.08.002 31668199

[9] : Lassa fever outbreaks in Nigeria. *Expert Review of Anti-infective Therapy* .2018;16(9) : 10.1080/14787210.2018.1512856 663-666 10.1080/14787210.2018.1512856 30111178

[10] : Lassa fever - the road ahead. *Nat Rev Microbiol* .2023;21(2) : 10.1038/s41579-022-00789-8 87-96 10.1038/s41579-022-00789-8 36097163 PMC9466315

[11] : Forty-two years of responding to Ebola virus outbreaks in Sub-Saharan Africa: a review. *BMJ Glob Health* .2020;5(3) : 10.1136/bmjgh-2019-001955 e001955 10.1136/bmjgh-2019-001955 32201623 PMC7061886

[12] : Lassa fever. Effective therapy with ribavirin. *N Engl J Med* .1986;314(1) : 10.1056/NEJM198601023140104 20-6 10.1056/NEJM198601023140104 3940312

[13] : Ribavirin for the treatment of Lassa fever: A systematic review and meta-analysis. *Int J Infect Dis* .2019;87: 10.1016/j.ijid.2019.07.015 15-20 10.1016/j.ijid.2019.07.015 31357056

[14] : Ribavirin for treating Lassa fever: A systematic review of pre-clinical studies and implications for human dosing. *PLoS Negl Trop Dis* .2022;16(3) : 10.1371/journal.pntd.0010289 e0010289 10.1371/journal.pntd.0010289 35353804 PMC9000057

[15] : Molecular diagnostics for lassa fever at Irrua specialist teaching hospital, Nigeria: lessons learnt from two years of laboratory operation. *PLoS Negl Trop Dis* .2012;6(9) : 10.1371/journal.pntd.0001839 e1839 10.1371/journal.pntd.0001839 23029594 PMC3459880

[16] : Lassa fever outcomes and prognostic factors in Nigeria (LASCOPE): a prospective cohort study. *The Lancet Global Health* .2021;9(4) : 10.1016/S2214-109X(20)30518-0 e469-e478 10.1016/S2214-109X(20)30518-0 PMC797045033740408

[17] : Lassa Fever among Children in Eastern Province, Sierra Leone: A 7-year Retrospective Analysis (2012-2018). *Am J Trop Med Hyg* .2020;104(2) : 10.4269/ajtmh.20-0773 585-592 10.4269/ajtmh.20-0773 33241780 PMC7866338

[18] : Pharmacokinetics of Ribavirin in the Treatment of Lassa Fever: An Observational Clinical Study at the Irrua Specialist Teaching Hospital, Edo State, Nigeria. *Clin Infect Dis* .2023;76(3) : 10.1093/cid/ciac578 e841-e848 10.1093/cid/ciac578 35881530 PMC9907520

[19] : Favipiravir and Ribavirin Treatment of Epidemiologically Linked Cases of Lassa Fever. *Clin Infect Dis* .2017;65(5) : 10.1093/cid/cix406 855-859 10.1093/cid/cix406 29017278 PMC5682919

[20] : A case-control study of the clinical diagnosis and course of Lassa fever. *J Infect Dis* .1987;155(3) : 10.1093/infdis/155.3.445 445-55 10.1093/infdis/155.3.445 3805772

[21] : Systematic review and meta-analysis of the epidemiology of Lassa virus in humans, rodents and other mammals in sub-Saharan Africa. *PLoS Negl Trop Dis* .2020;14(8) : 10.1371/journal.pntd.0008589 e0008589 10.1371/journal.pntd.0008589 32845889 PMC7478710

[22] : Factors associated with progression to death in patients with Lassa fever in Nigeria: an observational study. *The Lancet Infectious Diseases* .2021;21(6) : 10.1016/S1473-3099(20)30737-4 876-886 10.1016/S1473-3099(20)30737-4 33484646 PMC8212868

[23] : Viral hemorrhagic fevers: advancing the level of treatment. *BMC Med* .2012;10: 10.1186/1741-7015-10-31 31 10.1186/1741-7015-10-31 22458265 PMC3325866

[24] : Caring for critically ill patients with ebola virus disease. Perspectives from West Africa. *Am J Respir Crit Care Med* .2014;190(7) : 10.1164/rccm.201408-1514CP 733-7 10.1164/rccm.201408-1514CP 25166884

[25] : Moving Lassa Fever Research and Care Into the 21st Century. *J Infect Dis* .2017;215(12) : 10.1093/infdis/jix206 1779-1781 10.1093/infdis/jix206 28863471 PMC5853919

[26] : Roots, Not Parachutes: Research Collaborations Combat Outbreaks. *Cell* .2016;166(1) : 10.1016/j.cell.2016.06.029 5-8 10.1016/j.cell.2016.06.029 27368093 PMC5210219

[27] : The year that Ebola virus took over west Africa: missed opportunities for prevention. *Am J Trop Med Hyg* .2015;92(2) : 10.4269/ajtmh.14-0818 229-232 10.4269/ajtmh.14-0818 25561568 PMC4347318

[28] : Clinical features of Lassa fever in Liberia. *Rev Infect Dis* .1989;11 Suppl 4: 10.1093/clinids/11.supplement_4.s783 S783-9 10.1093/clinids/11.supplement_4.s783 2749109

[29] : Lassa fever in post-conflict sierra leone. *PLoS Negl Trop Dis* .2014;8(3) : 10.1371/journal.pntd.0002748 e2748 10.1371/journal.pntd.0002748 24651047 PMC3961205

[30] : Lassa fever in West Africa: evidence for an expanded region of endemicity. *Zoonoses Public Health* .2012;59 Suppl 2: 10.1111/j.1863-2378.2012.01469.x 43-7 10.1111/j.1863-2378.2012.01469.x 22958249

[31] : Understanding the cryptic nature of Lassa fever in West Africa. *Pathog Glob Health* .2017;111(6) : 10.1080/20477724.2017.1369643 276-288 10.1080/20477724.2017.1369643 28875769 PMC5694855

[32] : Central nervous system manifestations of lassa fever in Nigeria and the effect on mortality. *Journal of the Neurological Sciences* .2013;333: 10.1016/j.jns.2013.07.2107 10.1016/j.jns.2013.07.2107

# Response

**Published:** 2023-05-25

**Authors:** Josephine Bourner

**Affiliations:** University of Oxford, UK

Dear Dr Akpede,

 We would like to thank you for the generous feedback you’ve provided on the protocol. Based on your feedback we have made a number of clarifications and additions to the manuscript, and provided a point-by-point summary of our response in the PDF file linked here.

 We hope that our responses provide sufficient detail for your approval.

 Sincerely,

 Josephine Bourner

 On behalf of the West Africa Lassa fever Consortium (WALC): Work Package 2 working group

# Reviewer report: Reviewer response for version 1

**DOI:** 10.21956/wellcomeopenres.21110.r55701

**Published:** 2023-04-13

**Authors:** Emmanuel Bottieau

**Affiliations:** 1 Department of Clinical Sciences, Institute of Tropical Medicine, Antwerp, Belgium

The authors present a pre-positioned protocol in order to evaluate in a randomized controlled trial the safety and efficacy of much-needed multiple new interventions for the therapy of hospitalized/severe patients with Lassa fever. I would like first to congratulate the study group for undertaking this pivotal and difficult research initiative (for all reasons nicely elaborated in the rationale section). I am fully supportive of this multicentric platform trial, as well as its adaptive design, with the best therapy becoming the control arm.

 I agree with the statement of the many unknowns regarding disease outcome, although some recent studies have provided key information that could be carefully integrated already in the current trial design. As such, the multi-stage approach seems mainly based on this “lack of knowledge”, but data from at least one large prospective clinical study could accelerate this process, as basis for some reliable assumptions (LASCOPE study, see below). Also, it is a bit unclear whether the first year will be “only” observational or will already compare different study arms (and if so, which ones).

 Please find below my main comments and suggestions:

 I suggest to add in the Introduction/rationale some epidemiological information, regarding the countries with highest endemicity, as well as some brief context-specific data about the study sites which will likely participate to the trial (even if it is a pre-positioned protocol).  

 Some more detailed technical information would be welcome, especially regarding the control arm (which, I understood, would be ribavirin: please provide the selected/agreed upon dose and duration of the regimen) and on at least one intervention arm. I understand favipiravir is the first candidate, closest to clinical evaluation. By the way could you clarify whether randomization will start during the first stage?

 I also suggest to reformulate/clarify the secondary objective (page 4) “The secondary objective of this protocol is to evaluate the prevalence of complications associated with Lassa fever”. Not sure prevalence is the most appropriate wording, as we talk about a diseased population (Lassa patients). Also I think that what matters most, is the clinical difference should be made between the frequency of “complications” (still to define which ones) at presentation/inclusion and the incidence/emergence of additional complications during the course of the disease (which would be important elements for the composite primary endpoint, see next paragraph).  

 The key problem clearly resides indeed in the definition of the primary endpoint. I agree with the expert group that mortality is not sufficient as primary endpoint for a trial conducted in specialized research trial sites, as the authors demonstrate that a very large sample size would be needed for interventions with moderate effect. In addition, survival might further increase during the trial as supportive care “improves” and Lassa cases are detected earlier. It is proposed to define a composite primary endpoint which makes a lot of sense as end-organ failure usually results in death in low-resource settings. An endpoint “unfavorable outcome” had been already suggested in a recent publication (Olayinka *et al.*, 2022 ^1^). In this protocol, the following composite primary endpoint is proposed: mortality OR progression to severe disease, as defined by new onset of acute failure (KDIGO 3) OR new onset of shock OR new onset of acute respiratory failure.

 There are several questions regarding this key aspect:

 I miss some quantitative assumptions regarding the rate of new complications reflecting this “progression/deterioration” to end-organ failure. One important prospective clinical study (Duvignaud *et al.*, 2021 ^2^; 510 participants, all exposed to ribavirin) provides some good “baseline” estimations of both the frequency of such complications at inclusion/admission and more importantly the rate of new ones emerging during the course of the disease (usually within a few days). Looking at this publication, we get some interesting data, briefly summarized as follows:

- KDIGO 3: from 8% at inclusion to 12% thereafter (note that KDIGO 2 was also associated with death in this study), i.e. an increase of 4%
- hypoxemia (SaO2 < 92%) : from 7 to 15%, i.e. an increase of 8%)
- shock:  from 5% to 22%, an increase of 17%
- altered consciousness; from 6% to 14%, an increase of 8%; seizure from 2% to 5%
- bleeding; from 19 to 34%, and increase 15% (note that WHO events grade 2 to 4 were reported)

Based on this study, I miss first some rationale to explain why new-onset bleeding and new-onset encephalopathy could not be added to the composite primary endpoint. If it has been decided so after the expert discussion, it would be good to state it as such, and explain why.

 Also, looking at those rather solid data, I wonder whether some more detailed assumptions could not be made from the beginning of the trial, to avoid maybe the first “one-year stage”. Of course, what matters is the frequency of any new-onset complications (compared to the baseline/inclusion), so the frequency of the emergence of each complication in isolation has to be known (as they are likely often combined). This could probably be obtained by re-analyzing the LASCOPE dataset, maybe consolidated by additional (unpublished) information from other study sites. Following this line, and even if a single-center study represents a clear limitation (but also the best evidence we have so far) it would be worth estimating more robustly (1) the frequency of ANY end-organ failure at inclusion and (2) more importantly the combined rate of mortality and ANY new-onset complications (could 30% be a good guess estimate?). I suggest to add such a synoptic Table in the current protocol, and maybe to get some statistical re-calculation for -  say – an at least 30% relative reduction of the frequency of unfavorable outcome (from 30% to 20% for this example). Efficacy and futility rules could also be based on these calculations. Of course an interim analysis would remain important, to assess the reliability of those initial assumptions during the course of the trial and possibly readjust the sample sizes.

Is the study design appropriate for the research question?

Yes

Is the rationale for, and objectives of, the study clearly described?

Partly

Are sufficient details of the methods provided to allow replication by others?

Partly

Are the datasets clearly presented in a useable and accessible format?

Not applicable

Reviewer Expertise:

Medical doctor, specialized in Internal Medicine, Infectious and Tropical Diseases (clinician)

I confirm that I have read this submission and believe that I have an appropriate level of expertise to confirm that it is of an acceptable scientific standard, however I have significant reservations, as outlined above.

## References

[1] : A standardised Phase III clinical trial framework to assess therapeutic interventions for Lassa fever. *PLoS Negl Trop Dis* .2022;16(1) : 10.1371/journal.pntd.0010089 e0010089 10.1371/journal.pntd.0010089 34990453 PMC8769305

[2] : Lassa fever outcomes and prognostic factors in Nigeria (LASCOPE): a prospective cohort study. *The Lancet Global Health* .2021;9(4) : 10.1016/S2214-109X(20)30518-0 e469-e478 10.1016/S2214-109X(20)30518-0 PMC797045033740408

# Response

**Published:** 2023-05-25

**Authors:** Josephine Bourner

**Affiliations:** University of Oxford, UK

Dear Dr Bottieau,

 We would like to thank you for the generous feedback you’ve provided on the protocol. Based on your feedback we have made a number of clarifications and additions to the manuscript, and provided a point-by-point summary of our response in the PDF file linked here.  

 We hope that our responses provide sufficient detail for your approval.

 Sincerely,

 Josephine Bourner

 On behalf of the West Africa Lassa fever Consortium (WALC): Work Package 2 working group
